# Field phenotyping of ten wheat cultivars under elevated CO_2_ shows seasonal differences in chlorophyll fluorescence, plant height and vegetation indices

**DOI:** 10.3389/fpls.2023.1304751

**Published:** 2024-01-08

**Authors:** Oliver Knopf, Antony Castro, Juliane Bendig, Ralf Pude, Einhard Kleist, Hendrik Poorter, Uwe Rascher, Onno Muller

**Affiliations:** ^1^ Institute of Bio- and Geosciences: Plant Sciences (IBG-2), Forschungszentrum Jülich GmbH, Jülich, Germany; ^2^ INRES-Renewable Resources, University of Bonn, Rheinbach, Germany; ^3^ Department of Natural Sciences, Macquarie University, North Ryde, NSW, Australia

**Keywords:** CO_2_, wheat, fluorescence, phenotyping, climate change, senescence, chlorophyll, FACE (Free-Air CO_2_ Enrichment)

## Abstract

In the context of climate change and global sustainable development goals, future wheat cultivation has to master various challenges at a time, including the rising atmospheric carbon dioxide concentration ([CO_2_]). To investigate growth and photosynthesis dynamics under the effects of ambient (~434 ppm) and elevated [CO_2_] (~622 ppm), a Free-Air CO_2_ Enrichment (FACE) facility was combined with an automated phenotyping platform and an array of sensors. Ten modern winter wheat cultivars (*Triticum aestivum* L.) were monitored over a vegetation period using a Light-induced Fluorescence Transient (LIFT) sensor, ground-based RGB cameras and a UAV equipped with an RGB and multispectral camera. The LIFT sensor enabled a fast quantification of the photosynthetic performance by measuring the operating efficiency of Photosystem II (F_q_’/F_m_’) and the kinetics of electron transport, i.e. the reoxidation rates F_r1_’ and F_r2_’. Our results suggest that elevated [CO_2_] significantly increased F_q_’/F_m_’ and plant height during the vegetative growth phase. As the plants transitioned to the senescence phase, a pronounced decline in F_q_’/F_m_’ was observed under elevated [CO_2_]. This was also reflected in the reoxidation rates F_r1_’ and F_r2_’. A large majority of the cultivars showed a decrease in the harvest index, suggesting a different resource allocation and indicating a potential plateau in yield progression under e[CO_2_]. Our results indicate that the rise in atmospheric [CO_2_] has significant effects on the cultivation of winter wheat with strong manifestation during early and late growth.

## 1 Introduction

Since the industrial age, the atmospheric CO_2_ concentration ([CO_2_]) has increased by 50%, from 280 ppm at the end of the 19th century to 418 ppm in 2022 (Keeling et al., 1976; IPCC, 2021a; NOAA ESRL, 2023). Current climate scenarios predict a further increase to an expected level of 550 ppm CO_2_ by 2060 (IPCC, 2021b), resulting in significant global climatic changes, including rising air temperatures and changing precipitation patterns (IPCC, 2021a). The complex and variable responses of crops to elevated CO_2_ concentrations complicate crop management and breeding strategies, making it increasingly difficult to meet the food demands of a growing population (FAO, 2011; OECD and FAO, 2012).

Understanding genotype, environment, and management (GxExM) interactions and developing resource-efficient, climate-resilient crops are among the top priorities for plant scientists and breeders (Beres et al., 2020; Cooper et al., 2021). Plant breeding has evolved over the years by incorporating new tools and technologies, but the main objectives remain unchanged - improving crop productivity by selecting heritable traits (Reynolds and Braun, 2022). Given the dynamic nature of the effects resulting from increased atmospheric CO_2_, crops will need to adapt to an increasingly different growing environment within a few breeding cycles. Wheat plays a crucial role as the third most important staple crop, providing one-fifth of the global caloric intake. It also is a major source of income for many small-scale farmers, and therefore also a significant contributor to global economic development (FAO, 2021).

Since CO_2_ is a key molecule in the plants’ carbon assimilation process, increased atmospheric CO_2_ levels may also lead to notable increases in photosynthesis (Long et al., 2004). Improving the effectiveness at which crops capture and convert H_2_O, CO_2_ and light energy into substance, i.e. photosynthetic efficiency, is regarded as a key pathway to achieving our sustainable development goals and is expected to play a significant role in the Fourth Green Revolution (Long et al., 2015). The fundamental prerequisite is a better understanding of the light-use efficiency of crops under dynamic light conditions and their interactions with the environment. Studying the highly dynamic photosynthesis process under such conditions presents a challenge to overcome. To address this knowledge gap, a number of elaborate growth chamber experiments, open-top chambers and free-air CO_2_ enrichment (FACE) emerged after the publication of the Brundtland report in 1987 (United Nations, 1987). FACE experiments mimic future atmospheric CO_2_ conditions under actual field conditions. Among the most important findings of these experiments is the capability of elevated CO_2_ concentrations (e[CO_2_]) to boost photosynthetic assimilation rates and increase the productivity of C_3_ and C_4_ plants (Ainsworth and Long, 2004; Ainsworth and Long, 2021; Gardi et al., 2022). Despite these advances, it remains unclear whether we can fully capture and describe the physiologically relevant dynamic features of field photosynthesis in sufficient detail. Therefore, Murchie et al. (2018) emphasise the need for extensive field data collection at different time points over the growing season.

In response to the growing need for a more holistic quantitative assessment of plants, the last decade has seen a surge in advanced phenotyping platforms, as highlighted by Cendrero-Mateo et al. (2017). These incorporate automated imaging, robotics, and machine learning to analyse plant growth, physiology, and morphology on a large scale. Recent sensor advancements in remote sensing and field phenotyping have shifted the focus from individual plants to canopy and field-level observations. These non-invasive methods enable quicker, more accurate measurements of plant traits, greatly benefiting breeding and genetic studies.

RGB imagery and Chlorophyll Fluorescence (ChlF) acquisition methods have shown great promise in studying the impact of abiotic and biotic stress on various plant species and crops (Rebetzke et al., 2019; Fu et al., 2022; Pieruschka and Schurr, 2022). ChlF is a measurable optical signal resulting from the competing light energy pathways in plants where light is either (a) utilised in photosynthesis (photochemistry), (b) transferred to other pigments, (c) dissipated in the form of heat (NPQ) or (d) re-emit as a byproduct with a longer wavelength in the form of fluorescence. ChlF can be quantified using active instruments containing an excitation light source and a fluorometer (Maxwell and Johnson, 2000; Baker and Rosenqvist, 2004; Baker, 2008). Pulse-amplitude modulation (PAM) fluorometry is commonly used for active ChlF measurements, but its limitations, such as short-distance applicability, hinder large-scale open field studies with high throughput. To address these challenges, the Light-induced fluorescence transient device (LIFT) has emerged as a promising alternative to actively quantify ChlF traits, including the PSII operating light-use efficiency of light-adapted plants (F_q_’/F_m_’) (Osmond et al., 2017; Keller, 2018). Previous work has suggested that LIFT can be useful for uncovering genetic variation in response to environmental stress (Zendonadi dos Santos et al., 2021).

Several studies investigated the effect of e[CO_2_] on photosynthetic assimilation (Lauriks et al., 2021) and efficiency (Javaid et al., 2022) at different stages of development in diverse plant species yielding various outcomes – a large number of them were devoted to understanding long term effects of tree species and grasslands. A growth chamber experiment with *Acacia logifolia* showed increased photosynthetic assimilation per unit leaf area under e[CO_2_], mainly at the beginning of the growing period. As the growing period progressed, relative differences in assimilation under a[CO_2_] and e[CO_2_] got smaller and finally dropped significantly (*p* = .001) towards the end of the experiment (Javaid et al., 2022). Potential alterations of the photosynthetic efficiency under e[CO_2_] could be substantially relevant to global agricultural production. Most previous studies on the effect of CO_2_ enrichment on senescence reported either no changes or a delay in plant senescence (Curtis et al., 1989; Taylor et al., 2008). On the other hand, there are also studies which reported a slightly earlier flowering and senescence under e[CO_2_], often associated with the underlying concept that e[CO_2_] boosted photosynthesis; this ramped up sugar accumulation, depleting chloroplast nitrogen reserves faster and thus directly affecting the C/N balance resulting in oxidative stress (Agüera and De la Haba, 2018; Zani et al., 2020). A greenhouse experiment from Marc and Gifford (1984) investigated the floral initiation of wheat under e[CO_2_] and did not show an apparent effect on the crop. Bresson et al. (2018) suggest that senescence is not only driven by environmental factors but also genotypic properties, as well as the development of the plant. In turn, each of these factors can affect the onset, intensity and rate of progression of senescence. These previously observed species-dependent responses urge the need to study photosynthesis and the seasonal dynamics of crops under elevated CO_2_ closer.

This study introduces a pioneering approach by combining a LIFT instrument to monitor photosynthesis with an automated field phenotyping platform. This unique combination enables investigating winter wheat growth under elevated [CO_2_] in a typical agricultural field environment.

Our study aimed to employ novel techniques to gather a comprehensive dataset, enhancing our understanding of how e[CO_2_] influences the photosynthetic dynamics and growth patterns of winter wheat across its various developmental stages. In particular, we wanted to (1) provide a comprehensive description of the combination of a FACE system with an automated field phenotyping platform, emphasising its capabilities and contributions to the research, (2) assess the final biomass and various yield parameters across cultivars, (3) investigate seasonal growth dynamics by the help of UAV data, and (4) evaluate the seasonal variation in ChlF-related traits and the influence of abiotic factors, in response to ambient and elevated [CO_2_].

## 2 Materials and methods

### 2.1 Plant material and crop management

Ten modern winter wheat (*Triticum aestivum* L.) cultivars, released between 2014 and 2020 (**Appendix Table A1**), were evaluated within the ‘BigBaking project’. These cultivars, provided by nine European breeders, are commonly used in Germany. The selection targeted high-yielding cultivars with genotypic diversity from different quality groups to validate recent breeding efforts and to assess their resilience to climate change. Additionally, these cultivars served for subsequent baking quality and proteomics analysis to explore the relationship between yield and grain quality under e[CO_2_]. The cultivars were grown in plots sized 2 x 3 m with a sowing density of 330 kernels per m^-2^ and a row distance of 0.11 m. Winter wheat was sown on October 22nd 2020, with three replicate plots per treatment and in a complete randomised plot design. Plants emerged on November 2nd 2020 and were harvested on August 12th 2021. A total of 160 kg N per ha^-1^ liquid nitrogen fertilisers were applied in three doses of 60/60/40 kg per ha^-1^ on March 24th, April 20th and June 7th 2021. The field was managed following standard agricultural practices for the region and was monitored regularly to prevent damage from pests and pathogens. Significant crop management events were summarised together with phenological stages in **Table 1**.

**Table 1:** Important cultivation measures and relevant phenological stages.

Event	Phenological stage	Date	Day of Year	
Management				
Sowing		22-Oct-20	296	
	Emergence	02-Nov-20*	307	
Herbicide treatment (Malibu)		04-Nov-20	309	
Start of CO_2_ enrichment		13-Nov-20	318	
	Leaf development	16-Nov-20*	321	
1^st^ fertiliser application (AHL 30% N)		24-Mar-21	83	
Growth regulator (CCC, Moddus)		25-Mar-21	84	
	Canopy closure	30-Mar-21*	89	
	Stem elongation	15-Apr-21*	105	
2^nd^ fertiliser application (AHL 30% N)		20-Apr-21	110	
Fungicide and growth regulator (Input, Moddus)		23-Apr-21	113	
3^rd^ fertiliser application (AHL 30% N)		07-May-21	127	
Fungicide treatment (Ascra Xpro)		21-May-21	141	
	Heading	31-May-21*	151	
Fungicide and insecticide (Protendo, Solzil, Karate)		08-Jun-21	159	
	Anthesis	14-Jun-21*	165	
	Ripening	28-Jun-21*	179	
End of CO_2_ enrichment		12-Jul-21	193	
Harvest	Grain maturity	12-Aug-21	224	

*indicates the time point where a majority of the cultivars reached that stage.

### 2.2 Study site, experimental design, and phenotyping platform

The study was conducted at Campus Klein-Altendorf, the experimental field site of the University Bonn, near Rheinbach, Germany (50°37’ 29.3196” N 6°59’12.9834 “E, elevation: 177 m). Over the past 64 years, the mean annual temperature has been 9.6°C, and the mean annual precipitation was 603 mm year^-1^. Situated in the Lower Rhine Bay, the region is influenced by the Atlantic climate with prevailing westerly winds. The soil is classified as a Haplic Luvisol developed from loess with high clay content and high soil fertility (Pätzold and Pude, n.d).

**Figure 1:**
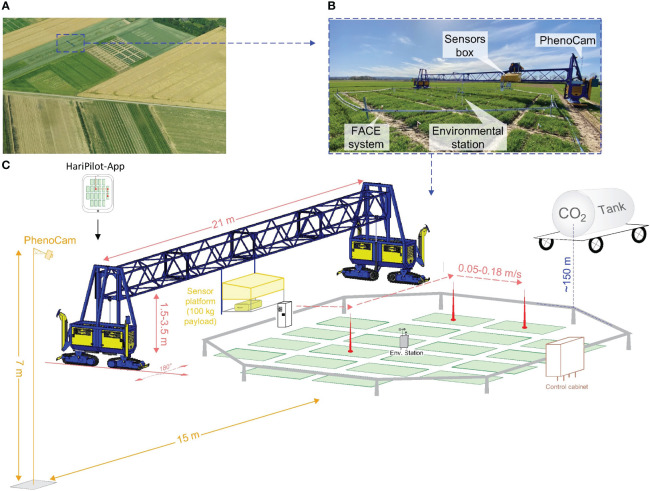
The Free-Air CO_2_ Enrichment (BreedFACE) experiment and the automated multipurpose phenotyping platform. **(A)** Aerial view of the BreedFACE field at Campus Klein-Altendorf, Rheinbach, Germany. **(B)** Ground-level photograph of the automated ‘FieldSnake’ phenotyping platform (shown in blue) alongside the FACE system. **(C)** A detailed illustration of the setup, highlighting the structural components such as the octagonal ring structure surrounding the winter wheat plots, the control cabinet, the CO_2_ supply tank, the environmental station, and PhenoCam alongside with the ‘FieldSnake’.

To elevate the atmospheric CO_2_ concentration (e[CO_2_]), a Free-Air Carbon Dioxide Enrichment (FACE) experiment was set up. This so-called BreedFACE facility is a mobile system and, therefore, allows for a three-year crop rotation system with winter barley (*Hordeum vulgare*) as a pre- and follow-crop (Muller et al., 2018; Quiros-Vargas et al., 2021; Soares et al., 2021). Eight ~7.25 m long steel pipes were joined together to form an octagonal structure of 18.5 m diameter (~254 m^2^). This sub-construction could be adjusted to the canopy height and was used to attach smaller poly-vinyl-chlorid pipes with tiny holes every 20 cm where pure CO_2_ was released. The amount of released CO_2_ was adjusted in real-time depending on the wind direction, wind speed and the actual CO_2_ concentration. Those factors were measured at a centrally positioned environment station together with the photosynthetic active radiation (model LI-190R, Li-COR Inc., Lincoln, NE, USA, licor.com/env/products/light/quantum), air temperature and air humidity. The target [CO_2_] was set to 600 ppm and measured using different CO_2_ sensors (HMP110, GMP343 and GMT221, Vaisala Oyj, Vantaa, Finland, vaisala.com). Over the growing season, the sensors were adjusted so they were always slightly above the canopy, i.e. a minimum of 0.2 m higher (**Figure 1**).

**Figure 2:**
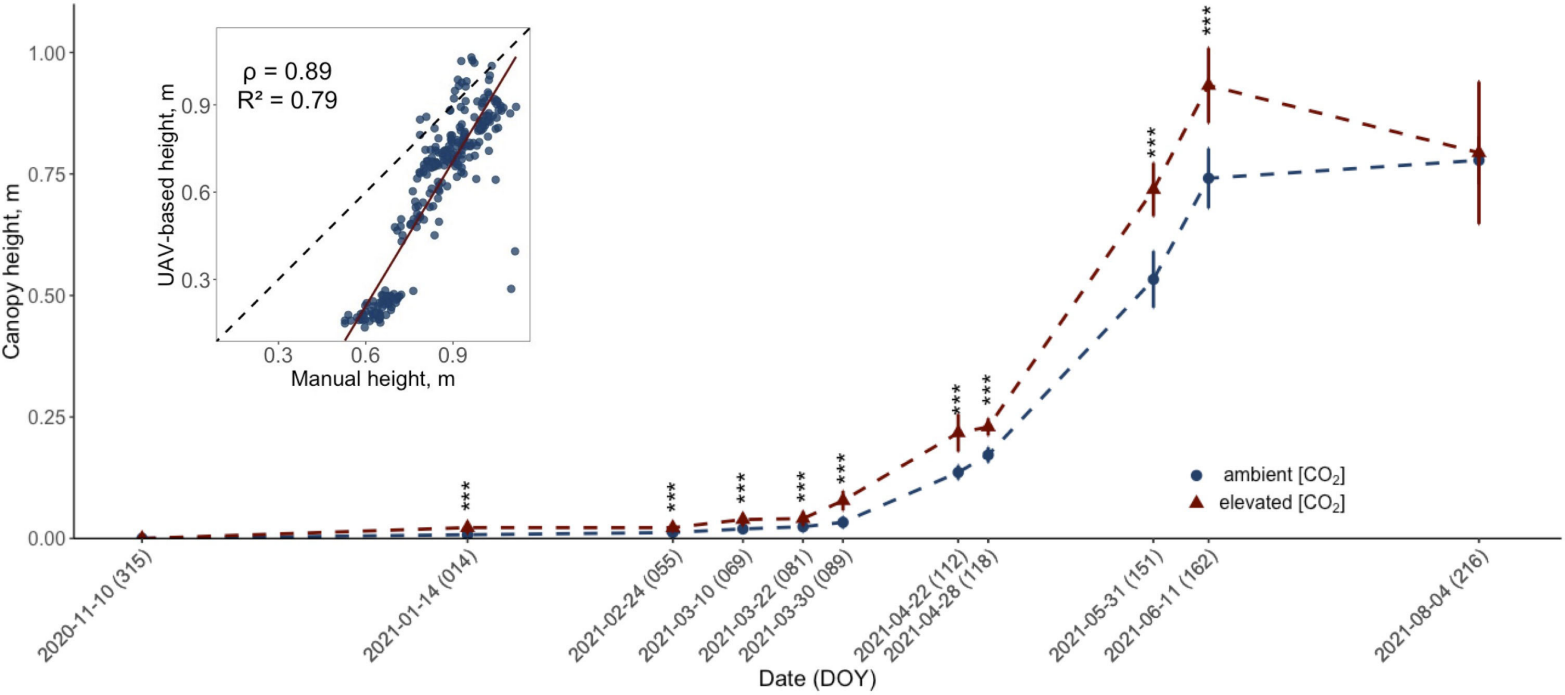
Development of the UAV-retrieved canopy height (m) for ten modern winter wheat cultivars (*Triticum aestivum* L.) grown under ambient (~434 ppm) and elevated (~622 ppm) [CO_2_] throughout the 2020/2021 vegetation period. The data for each treatment group is pooled across cultivars. Plants were cultivated in the BreedFACE experimental field at Campus Klein-Altendorf, Rheinbach, Germany. Error bars indicate the standard deviation of the mean height. Statistical significance was assessed using Welch’s Two Sample t-test, *p*< 0.001 *** (*n* = 30). The insert in the figure shows the correlation between manual and UAV-based measurements, with the correlation coefficient (ρ) and coefficient of determination (R^2^).

CO_2_ feeding started with plant emergence in November 2020, was paused for two weeks for safety reasons over New Year and was elevated with a few minor interruptions as long as we could perform measurements with detectable photosynthesis activity. This was the case until July 12th, 2021. As day length increased throughout the season, the CO_2_ feeding period was adapted, ranging from 9 hours per day during the early vegetation period up to 13 hours per day later in the year.

The BreedFACE facility is complemented by the FieldSnake (see **Figures 1B, C**), a semi-automated mobile phenotyping platform (prototype developed by Lommers Tuinbouwmachines, Bergeijk, The Netherlands and the Forschungszentrum Jülich, Jülich, NRW, Germany). Integral part of the FieldSnake is a movable measurement platform that is attached to a bridge, adjustable in height (1.5 – 3.5 m) and capable of carrying various phenotyping sensors up to a payload of 100 kg. The 20-meter-wide bridge is supported by compartments on each side (i.e. engine and steering) running on caterpillars.

Under human surveillance, the FieldSnake is capable of navigating autonomously over the experimental field at a speed of about 2-3 km/h with the help of three Global Navigation Satellite System (GNSS) antennas, two of them positioned at the outer edges of the machine and one directly on the measurement platform. The GNSS signal is supplemented by the German SAPOS reference service (https://sapos.de/) to optimise the accuracy of the navigation to centimetres. The data acquisition pattern was set beforehand in an iOS-based mobile application (HariPilot, Hari Tech KFT, Pötréte, Hungary, https://hari-tech.hu/) and could be checked and adapted on an iPad among other settings, e.g. the traverse speed of the measurement platform (0.05 – 0.18 m/s), measurement height, acquisition mode (scanning or stationary). Positioning data were logged every second, together with other relevant parameters and transmitted to a server.

### 2.3 LIFT and PhenoCam data acquisition

A Light-Induced Fluorescence Transient device (model LIFT-REM 1.0, Soliense Inc., Shoreham, NY, USA; https://soliense.com/LIFT_Terrestrial.php) was employed to monitor variations in ChlF. The active probing method first described by Kolber et al. (1998) induces chlorophyll fluorescence by emitting a series of sub-saturating excitation light impulses at a fast repetition rate (FRR). Multiple lenses focus the light of a blue (λ 450 nm) light-emitting diode (LED) to a 40 mm light beam at a distance of 0.6 m. By the FRR method, the capacity of Electron Transport to Photosystem II (PSII) is exceeded, causing reaction centres to close. Thereby, resulting changes in the plants’ fluorescence signal can be measured within microseconds by an avalanche photodiode in combination with an optical interference filter (685 nm ± 10 nm) that separates the red ChlF from the rest of the light. The device was programmed to progressively saturate the plastoquinone A pool (S_QA_ sequence) within 300 ms. Following the maximum ChlF (F_m_'), a 127 ms long relaxation sequence (R_QA_) with exponentially increasing breaks between the flashlets allows Q_A_ to reoxidate (Osmond et al., 2017). This saturation-relaxation-measurement protocol ultimately results in a transient of which kinetics can be calculated (**Appendix Figure A1**). The PSII operating efficiency for light-adapted plants (F_q_'/F_m_') has been found to exhibit a high correlation with F_v_'/F_m_' measurements obtained using pulse amplitude modulation (PAM) techniques (Wyber et al., 2017). F_r1_' and F_r2_' are parameters used to characterise the electron transport kinetics of light-adapted plants during the R_QA_ sequence, as described by Zendonadi dos Santos et al. (2021). These parameters are obtained through log-log-transformed regression analysis of the slope of the transient, and they correspond to two specific time intervals of the measurement protocol, i.e. t_1_ 0.82-1.44 ms and t_2_ 1.56-8.08 ms. Within these intervals, electron transfer occurs from Q_A_ to the PQ pool and, to some extent, from the PQ pool to PSI.

To closely follow the senescence period, a permanent setup of two identical RGB cameras (model SD500BN, StarDot Technologies, Buena Park, CA, USA) was set up at a fixed distance from the ring (15 m) to ensure continuous measurements of phenological changes (PhenoCams). They were mounted on a tower of 7 m in height and acquired daily images from June 13th till the harvest on August 12th, 2021 (**Figure 1**).

### 2.4 Field measurements

#### 2.4.1 LIFT measurements

During the vegetation period, nine LIFT measurements were conducted bi-weekly under clear-sky conditions (see **Appendix: Table A2**), starting with canopy closure from late March (DOY 89) to mid-July (DOY 193). Therefore, the 15 kg heavy LIFT instrument capable of measuring ChlF up to a distance of 3 m was mounted to the FieldSnake platform, levelled by a gimbal pointing downward (Nadir) at a fixed measurement distance of ~0.6 m above the canopy. The platform’s height was adjusted to match the average canopy height of the plants grown at a[CO_2_] and e[CO_2_]. Scanning at approximately 10 cm s^-1^ along crop rows, over 1’200 point measurements were acquired across the BreedFACE field, with around 20 measurements per plot.

#### 2.4.2 Manual height measurements

Canopy height was assessed manually six times throughout the vegetation period by measuring the vertical distance from the tip of the plant closest to the ruler to the soil level. These measurements were performed using a folding ruler at three random locations across the plots.

#### 2.4.3 UAV data acquistion

**Figure 3:**
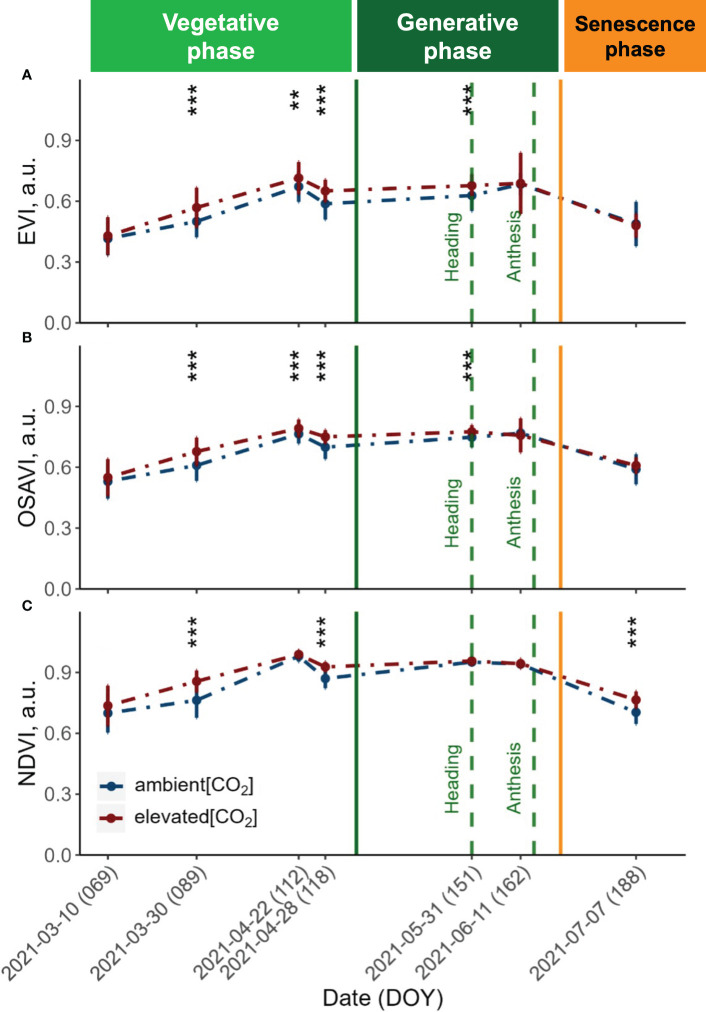
Seasonal dynamics of the **(A)** Enhanced Vegetation Index (EVI), **(B)** Optimised Soil-Adjusted Vegetation Index (OSAVI) and **(C)** Normalised Difference Vegetation Index (NDVI) derived from UAV with a Mica-Sense camera. Pooled data of ten modern winter wheat cultivars (*Triticum aestivum* L.) grown under ambient (~434 ppm) and elevated (~622 ppm) [CO_2_] measured throughout the vegetation period 2020/2021. Plants were grown in the BreedFACE experimental field at Campus Klein-Altendorf, Rheinbach, Germany. Error bars indicate the standard deviation from the mean. ** p<.01 *** p<001.

A total of twelve flight campaigns covered key growth stages of the crop from November 2020 to July 2021 (**Figure 3**). Flights were mostly conducted under clear-sky conditions around midday (see **Appendix: Table A2**). To obtain RGB and multispectral data, a Sony ILCE-7RM3 respectively a MicaSense dual camera system (AgEagle Sensor Systems Inc., Wichita, KS, USA) were mounted to an Unmanned Aerial Vehicle (UAV, DJI Matrice Pro 600, SZ DJI Technology Co., Ltd., Shenzhen, China) as described in Chakhvashvili et al. (2022). Geotagging was performed using onboard equipment. Flights were conducted at a 20 m altitude, resulting in 0.001-0.002 m pixel size.

#### 2.4.4 Harvest and post-processing

The above-ground biomass was determined at the harvest on August 12th 2022. A special combine harvester (model Quantum Plus, Wintersteiger AG, Ried, Austria) allowed for a core harvest of 1.5 x 2 m and separated grains from the shoot. The straw was immediately collected and weighed. Straw sub-samples were taken, weighed, and dried for 72 h in a drying oven at 70°C before being weighed again to calculate the vegetative dry matter. Grain samples underwent additional cleaning, and their weight, along with the thousand-grain weight, was measured before conducting subsequent analysis.

### 2.5 Data processing and statistics

#### 2.5.1 LIFT and FieldSnake data processing

Data were mainly processed using R (Version 2022.02.2 + 485, R Core Team, 2013). After processing the acquired LIFT raw data, the data was linked to spatial data logged by the FieldSnake using timestamps from both systems. The combination of data allowed to correct for the sensor mounting offset, enabling further spatial data cleaning in QGIS (Version 3.24.2), e.g. excluding measurements in border areas (30 cm).

#### 2.5.2 UAV data processing

Digital elevation models (DEMs) were generated from RGB imagery and orthomosaics from multispectral imagery using AgiSoft Metashape (Version 2.0.1). Nine panels with varying reflectance factors were used for processing multispectral images to top of canopy reflectance (Chakhvashvili et al., 2022). We chose to analyse the Normalised Difference Vegetation Index (NDVI), a commonly used index for assessing vegetation health and cover. The Enhanced Vegetation Index (EVI), which offers refinements of the NDVI such as atmospheric correction and improved performance in densely vegetated areas, as well as the Optimised Soil-Adjusted Vegetation Index (OSAVI), which is used to minimise the impact of soil background (**Table 2**).

**Table 2:** Visible (VI_RGB_) and near-infrared vegetation indices (VI_NIR_) used in this study.

VI	Name	Formula	Reference
ExG	Excess Green Index	$\text{ExG }=2*\frac{\text{Green}}{\text{Red}+\text{Green}+\text{Blue}}-\frac{\text{Blue}}{\text{Red}+\text{Green}+\text{Blue}}-\frac{\text{Red}}{\text{Red}+\text{Green}+\text{Blue}}$	(Sonnentag et al., 2012)
EVI	Enhanced Vegetation Index	$\text{EVI }=2.5*\frac{\text{NIR}-\text{Red}}{\text{NIR}+6\text{ x Red}-7.5\text{ x Blue}+1}$	(Huete et al., 2002)
OSAVI	Optimised Soil-Adjusted Vegetation Index	$\text{OSAVI }=\frac{\text{NIR}-\text{Red}}{\text{NIR}+\text{Red}+0.16}$	(Rondeaux et al., 1996)
NDVI	Normalised Difference Vegetation Index	$\text{NDVI }=\frac{\text{NIR}-\text{Red}}{\text{NIR}+\text{Red}}$	(Peñuelas et al., 1994)

Plant height was extracted by subtracting DEMs from a respective growth stage from a DEM of bare soil (Bendig et al., 2014). Plot-level information was extracted using the zonal statistics function exact_extract (Baston, 2022). Data were further analysed and visualised in R.

#### 2.5.3 PhenoCam data processing

Within each image retrieved from the PhenoCam, a region of interest (ROI) with multiple points of interest was set for the cores of all plots. Values retrieved from the images were then normalised (Richardson et al., 2018) in order to calculate the excess green index (ExG, **Table 2**), which was used to describe plant senescence on a canopy level. The data was then fitted with a log-regression model for the time intervals between the LIFT measurements during the senescence period, i.e. senescence period 1 being DOY 168 – DOY 176 and senescence period 2 from DOY 176 to DOY 193 where the dependent PSII operating efficiency was log-transformed.

## 3 Results

### 3.1 Abiotic environmental parameters

At the experimental site, the mean temperature during the growing period (October 2020 - August 2021) was 10.1°C and thus half a degree warmer than the observed long-term average. While November 2020 was a rather cold month (-2.2°C), the months of February and June were relatively warm, with monthly mean temperatures deviating by +2.4°C and +3.2°C, respectively, compared to the long-term average. The annual precipitation reached 707 mm, representing a 17.3% increase compared to the recorded long-term average. This increase was mainly driven by heavy rainfall events in January and July 2021, during which more than twice the average amount of rainfall was recorded. With an annual sum of 1054 kWh m^-2^, the global radiation was slightly lower compared to the long-term measured global radiation of 1093.0 kWh m^-2^ per year.

Regarding the CO_2_ concentrations, the mean ambient CO_2_ concentration during operating hours was 434 ± 24 ppm in control plots and elevated by 43% to a mean concentration of 622 ± 57 ppm CO_2_ in the FACE system. During operating hours, the CO_2_ target concentration of 550 ppm and higher was reached 93% of the time at the ring’s centre (see **Appendix: Figure A2**).

### 3.2 Yield parameters

Given that intermediate destructive harvests were not feasible due to the limited number of replicates, plant height served as a proxy for seasonal biomass accumulation in this study. Following the start of the flight campaign in November 2020, a significant (*p*<.001) difference in plant height for winter wheat grown under e[CO_2_] was observed in January 2021 (DOY 14), with a mean difference of 1.43 cm ± .09 cm (**Figure 2**). This significant increase in plant height gradually continued throughout the entire vegetation period and peaked on June 11th, 2021 (DOY 162), with a mean difference of 19.11 cm ± 5.82 cm. However, on August 4th, 2021 (DOY 216), just before the harvest, a decline in canopy height was observed for plants grown under e[CO_2_].

To validate the UAV data, manual measurements were taken on six days from May 22nd, 2021 (DOY 142), to July 23rd, 2021 (DOY 204). These measurements confirmed the trend observed from the UAV data and showed a high correlation (ρ = .89, R^2^ = .79) with similar statistically significant differences. However, the difference in height between winter wheat cultivars treated with e[CO_2_] and the control was smaller. The maximum mean difference was measured on June 16th, 2021 (DOY 167), with plants under e[CO_2_] being 8.49 ± 3.08 cm taller, and a smaller decrease in mean height difference after that date to 7.07 ± 2.38 cm was recorded on July 23rd, 2021 (DOY 204). A more detailed look at the data revealed that while there were cultivar-specific variations in the extent of height increase, the overall trend — an increase in height in response to elevated CO_2_ levels — was consistently observed across all ten cultivars.

The relative difference in biomass was determined by the absolute weight at the end of the vegetation period, where significant CO_2_ effects were observed with notable increases in plants grown under e[CO_2_] compared to those grown under a[CO_2_]. Winter wheat cultivars grown under e[CO_2_] exhibited a significant (*p<*.001) increase in vegetative biomass, i.e. straw only (VDM, see **Table 3**). The mean vegetative biomass for plants grown under a[CO_2_] was 776.03 ± 59.16 g per m^2^, while plants grown under e[CO_2_] showed a 21.73% boost in vegetative biomass accumulation, reaching 936.86 ± 88.46 g per m^2^ (excluding two samples from Moschus due to a combine harvester processing failure). In terms of vegetative biomass, the cultivar Apostel displayed the strongest CO_2_ effect size of.28, with a 32.9% increase. In contrast to that, KWS Emerick experienced the smallest biomass increase of 16.3% under e[CO_2_].

**Table 3:** Relative change of various yield parameters.

Cultivar	VDM	GDM	TDM	HI	TGW	GN
Apostel	32.9 %***	6.2 %	17.3 %*	-9.7 %*	-7.8 %**	15.9 %*
Asory	26.3 %**	11.0 %	17.3 %**	-5.4 %	-7.7 %**	20.2 %**
Campesino	18.8 %*	20.8 %***	20.0 %**	0.8 %	-6.5 %*	29.5 %***
Foxx	17.9 %*	5.7 %	11.1 %	-4.9 %	-6.5 %*	12.7 %*
Hyvega	23.4 %**	-2.5 %	8.1 %	-9.8 %*	-8.1 %**	6.2 %
Informer	18.4 %*	6.7 %	11.7 %	-4.5 %	-2.9 %	9.9 %
KWS Emerick	16.3 %*	0.2 %	7.1 %	-6.3 %	0.7 %	-0.4 %
LG Initial	19.2 %*	6.0 %	11.6 %	-4.8 %	-7.4 %*	14.5 %*
Moschus	21.7 %*	4.4 %	11.2 %	-7.4 %**	-3.2 %	7.9 %
RGT Reform	22.3 %**	10.6 %*	15.6 %**	-4.4 %	-1.3 %	12.3 %*
Mean	21.7 %	6.9 %	13.1 %	-5.6 %	-5.1 %	12.9 %
Observations	60	62	60	60	62	62
Cultivar	0.138	**0.002**	**0.017**	0.171	**<0.001**	**<0.001**
e[CO_2_]	**<0.001**	**<0.001**	**<0.001**	**0.027**	**<0.001**	**<0.001**
Cult. x e[CO2]	0.97	0.252	0.201	**0.045**	0.208	0.063

Relative change of Vegetative Dry Matter (VDM), Grain Dry Matter (GDM), Total Dry Matter (TDM), Harvest Index (HI), Thousand-Grain Weight (TGW) and Number of Grains (GN) of plants grown under elevated CO_2_ and compared to ambient CO_2_. A two-way ANOVA was conducted to examine the effects of cultivars and elevated CO_2_ on yield components with Bonferroni-adjusted p-values shown below. Stars indicate statistically significant differences in the main effects analysed by pairwise comparisons, * p <.05 ** p<.01 *** p<.001.Bold values are significant at p < .05.

For grain yield (GDM), generally, smaller effect sizes were observed, but a significant treatment effect (*p*<.001) was documented. Harvesting the core area of the plots resulted in an average grain yield of 1017.23 ± 66.9 g per m^2^ for cultivars grown under a[CO_2_] and a mean yield increase of 7.6%, equivalent to 1094.59 ± 102.65 g per m^2^, for cultivars grown under e[CO_2_]. Although grain yield was generally positively affected by e[CO_2_], the response varied significantly among cultivars. Campesino exhibited a significant increase (*p*<.001) of 20.8% under e[CO_2_], while many other cultivars demonstrated weaker and non-significant responses.

Hyvega was the only cultivar that experienced a 2.5% decrease in grain yield under e[CO_2_]. Differences were also observed in Total Dry Matter (see TDM, **Table 3**), with an average increase of 13.10% in cultivar biomass under e[CO_2_]. Four cultivars showed a significant (*p*<.001) increase in biomass, with the highest increase of 19.95% observed in the cultivar Campesino.

The Harvest Index (HI, see **Table 3**) generally decreased, except for the cultivar Campesino, where the ratio between grain yield and vegetative mass remained consistent and was equally boosted under e[CO_2_]. The most significant shift in biomass accumulation was observed in Hyvega, where the HI decreased significantly (*p*<.05) by 9.79% under e[CO_2_].

The thousand-grain weight (TGW, see **Table 3**) decreased by 5.06%, with significant changes observed in six cultivars. Only KWS Emerick maintained its grain weight but, in turn, showed a slight decrease in the number of grains per m^2^. The number of grains increased in all other cultivars, with an average increase of 12.87% for cultivars grown under e[CO_2_]. Campesino displayed the most significant increase, with a 29.47% difference (see TGW, **Table 3**).

### 3.3 Phenology

The vegetation indices (VIs) obtained from the UAV-MicaSense setup revealed a comparable trend across all three indices throughout the observation period (**Figure 2**). Before the canopy closure on March 30th 2021, both the Enhanced Vegetation Index (EVI) and the Optimised Soil-Adjusted Vegetation Index (OSAVI) exhibited similarly low values, indicating a sparse and young vegetation cover. In contrast, the Normalised Difference Vegetation Index (NDVI) displayed increased values during the first flight campaign and continued to show consistently higher values over the entire vegetation period.

At canopy closure (DOY 89), all three VIs were significantly increased under e[CO_2_] (*p*<.001). This rise is indicative of an increase in vegetation biomass and greenness, reflecting the maturation and densification of the plant canopy. The values continued to rise until reaching their peak on April 22nd 2021, reflecting optimal vegetation health and productivity. Following this peak, a drop in all three VIs was observed, but values remained significantly higher (*p<*.001) for plants grown under e[CO_2_] until shortly after heading. At this stage, the NDVI did not exhibit any significant differences (*p* = 0.05) for plants grown under e[CO_2_]. Towards the end of the vegetation period, the three VIs displayed distinct patterns. While the EVI exhibited a trend of slightly decreased values under e[CO_2_], the NDVI showed the opposite effect, with values increasing under e[CO_2_].

**Figure 4:**
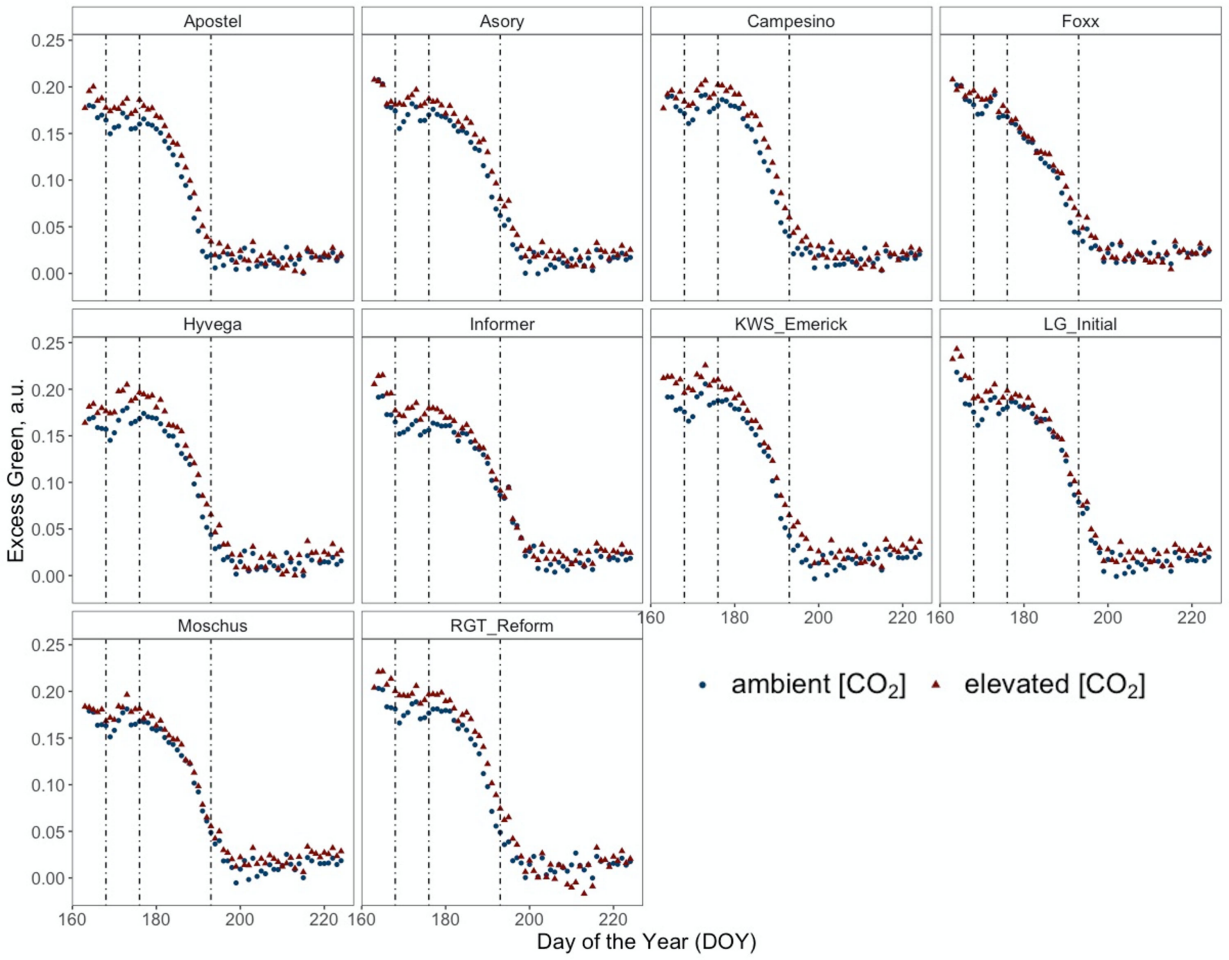
Normalised Excess Green index (ExG) retrieved from RGB images of PhenoCams monitoring the experiment during the senescence period from DOY 167 to DOY 225 of ten modern winter wheat cultivars (*Triticum aestivum* L.) grown under ambient (~434 ppm) and elevated (~622 ppm) [CO_2_] measured throughout the vegetation period 2020/2021. Plants were grown in the BreedFACE experimental field at Campus Klein-Altendorf, Rheinbach, Germany. Each dot represents the mean value of three plots (*n* = 3), and vertical lines indicate LIFT measurement dates and different senescence periods.

**Figure 5:**
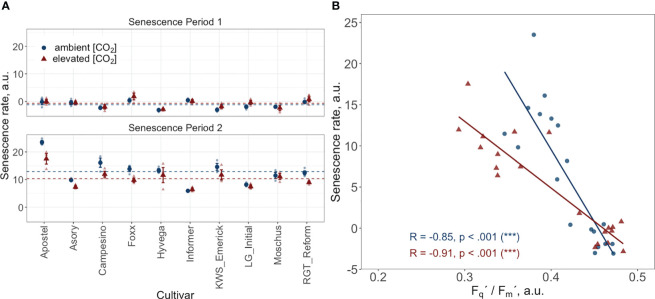
**(A)** Senescence rate during two periods: period 1 spans from the start of the grain filling phase, DOY 168 to DOY 176, and period 2 covers DOY 176 to DOY 193. Data points represent the mean senescence rate for each cultivar, with error bars indicating the standard error. Dashed horizontal lines indicate the overall mean senescence rate across all cultivars for the treatment and control groups during their respective periods. **(B)** Correlation between the PhenoCam-retrieved senescence rate and the LIFT-retrieved PSII operating efficiency (F_q_’/F_m_’) at two time points (DOY 168 and DOY 176) during the senescence phase. Data points represent the mean value per cultivar. Both datasets come from ten modern Winter wheat cultivars (*Triticum aestivum* L.) grown under ambient (~434 ppm) and elevated (~622 ppm) [CO_2_] in the BreedFACE experimental field at Campus Klein-Altendorf, Rheinbach, Germany, during the 2020/2021 vegetation period.

**Figure 4** presents a temporal high-resolution view of the plant senescence progression obtained by PhenoCams. Data retrieved from the PhenoCams indicate that the Excess Green index (ExG) was generally higher under e[CO_2_]. Whereas senescence duration is prolonged in cultivars like Apostel and Foxx, the onset is delayed in other cultivars such as Hyvega or RGT Reform and then progresses faster. In most cultivars, the e[CO_2_] treatment led to a further delay of senescence, which is also compensated in several cultivars by a faster progression rate and, in some cultivars, resulted in an even stronger degradation of chlorophyll according to the ExG index. According to PhenoCam data, plants grown under a[CO_2_] exhibited a higher senescence rate, i.e. the slope of the curve was steeper under a[CO_2_] in the second senescence period (**Figure 5A**).

The correlation between the LIFT-retrieved PSII operating efficiency and the PhenoCam-retrieved senescence rate was investigated at two independent time points during the senescence (DOY 168 and DOY 176). The findings show a significant negative relationship between the senescence rate and F_q_'/F_m_' at both time intervals. Plants grown under e[CO_2_] exhibited a correlation coefficient of -0.91, while plants grown under a[CO_2_] had a correlation coefficient of -0.85. These results suggest that an increase in senescence rate leads to a decrease in F_q_'/F_m_' or vice versa (**Figure 5B**).

### 3.4 Chlorophyll fluorescence traits

**Figure 6:**
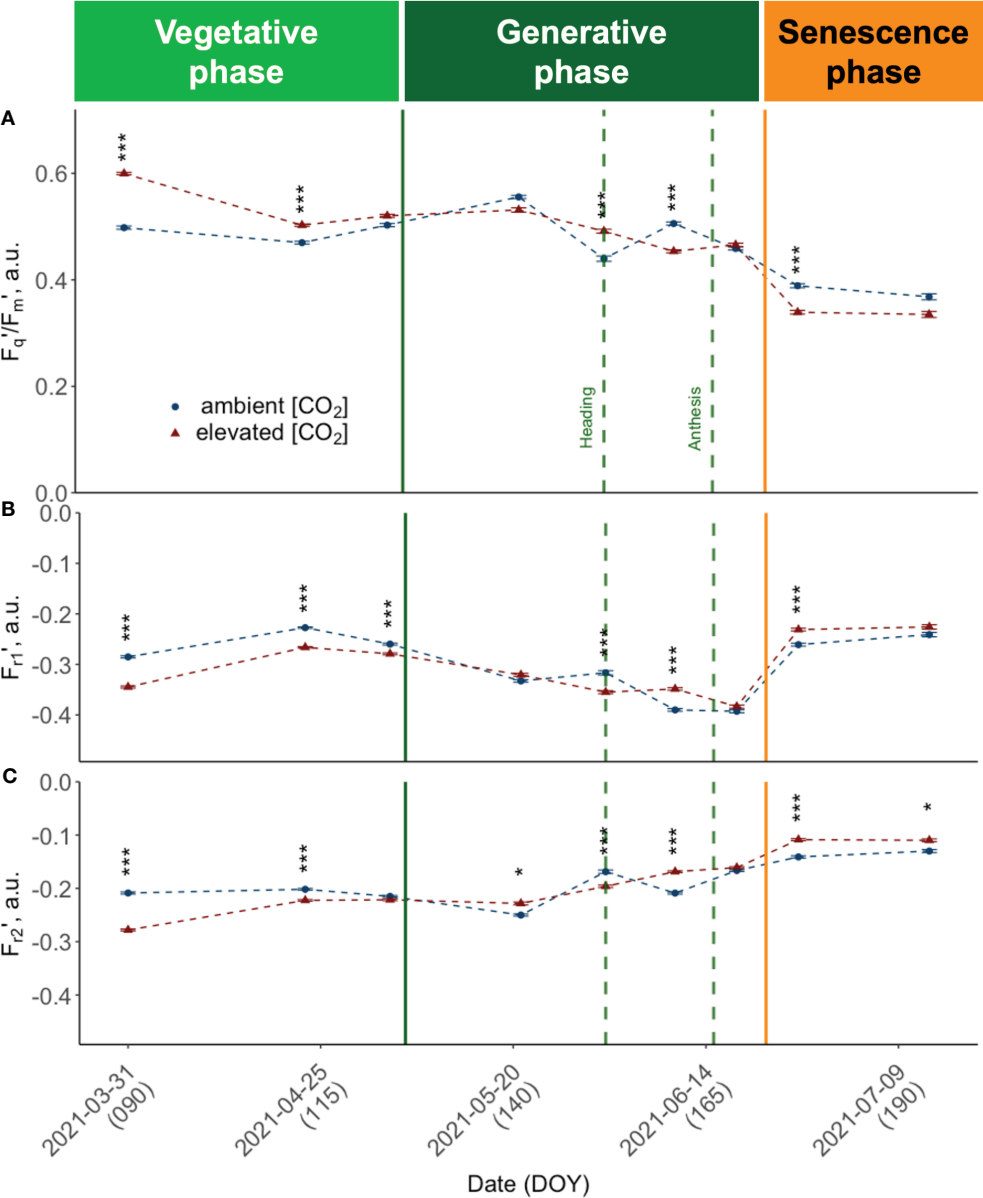
Seasonal dynamics of chlorophyll fluorescence traits, pooled data of ten modern light-adapted winter wheat (*Triticum aestivum* L.) cultivars grown under ambient (~434 ppm) and elevated (~622 ppm) [CO_2_]. **(A)** PSII operating efficiency (F_q_’/F_m_’) and **(B)** F_r1_’ representing the kinetics of electron transfer from QA to PQ pool, up to ~0.65 ms after F_m_’ is reached, i.e., F_r1_’ the kinetics of electron transfer from Q_A_ to PQ pool from light-adapted plants and **(C)** F_r2_’ the kinetics of electron transfer from PQ pool to PSI up to 6.64 ms after initiation of the measurement protocol. The data was collected using a LIFT-REM device in 2021 at the BreedFACE experimental field at Campus Klein-Altendorf, Rheinbach, Germany. Cultivar independent comparison, error bars indicate the SE, Bonferroni adjusted t-test, ns = not significant, *** p< 0.001 (*n* = 30, total number of measurements = 8’901). * p <.05.

Canopy closure in late March (DOY 89) marked the time point where it was possible to conduct consistent and reliable LIFT measurements. At this early vegetative growth stage, we observed consistently high PSII operating efficiencies (F_q_'/F_m_') across cultivars (**Figure 6A**). All cultivars grown under e[CO_2_] had higher F_q_'/F_m_' values compared to plants grown under a[CO_2_]. At this time, the retrieved values showed the highest absolute and relative differences between elevated and a[CO_2_]. Measured F_q_'/F_m_' was more than 20% higher under e[CO_2_] in cultivars such as Informer (*M* = 21.05%, *RE* = 1.60), Foxx (*M* = 20.34%, *RE* = 1.68) and Campesino (*M* = 20.16%, *RE* = 1.36). In order to investigate the effect of the three different growth periods, i.e. vegetative period (DOY< 125), generative period (DOY 125 - 160) and senescence period (DOY > 160) on the response variable (Fq'/F_m_'), a two-way ANOVA was conducted. The results showed no significant main effect of the CO_2_ treatment (F(1, 538) = .49, *p* = .484), but a highly significant main effect of the three different growth periods (F(2, 538) = 364.89, *p*<.001) and a highly significant interaction effect between CO_2_ and the growing period (F(2, 538) = 29.32, *p*<.001). The simple main effects were analysed to further investigate the nature of this interaction. The analysis of the main effects on F_q_'/F_m_' revealed a significant treatment effect (F(1, 184) = 53.91, *p*<.001) for the vegetative growth period. While mean values for the vegetative period were higher under e[CO_2_] (.531 ± .077) compared to a[CO_2_] (.484 ± .079), this changed during the generative period. There, winter wheat plants tended to have a higher mean F_q_'/F_m_' under ambient conditions (*M* = .491 ± .081 vs *M* = .505 ± .079). During the generative growth period, the analysis indicated no significant treatment effect (F(1, 183) = .42, *p* = .517). During the senescence period, mean values have dropped significantly to *M* = .394 ± .0969 for wheat cultivars grown under e[CO_2_] compared to *M* = 0.415 ± .0805 at a[CO_2_]. The simple main effects analysis of F_q_'/F_m_' did reveal a trend towards lower values under e[CO_2_] with a marginally significant treatment effect (F(1, 171) = 3.71, *p* = .056) due to larger variance during this final growth stage. On DOY 176, Campesino grown under e[CO_2_] had a by 30.66% lower mean F_q_'/F_m_' (*RE* = 2.19) than plants grown at ambient conditions. Overall, the results suggest that the CO_2_ treatment affected the operating efficiency of PSII predominantly during vegetative growth with reduced effect during later growth stages.

F_r1_’ and F_r2_' show a comparable trend (**Figures 6B, C**) that consistently opposes the behaviour observed in F_q_'/F_m_', resulting in a moderate to strong negative correlation (*r* -.58, respectively *r* = -.97). Specifically, at the beginning of the vegetation period, both F_r1_' and F_r2_' demonstrate higher values under a[CO_2_] conditions compared to e[CO_2_], with the difference between the two being more pronounced in the case of F_r2_'. As the seasons advanced, both F_r1_' and F_r2_' showed a declining trend. But while F_r2_' values increased again before heading, F_r1_' was decoupled, and values did not rise again before anthesis. Also for F_r1_' and F_r2_', a significant growth period effect (F(2,538) = 79.65, *p<* 0.001; F(2,538) = 364.89, *p<*.001) and interaction effect was documented (F(2,538) = 12.15, *p<*.001; F(2,538) = 29.32, *p<*.001). The analysis of the simple main effects resulted in no significant treatment effect for F_r1_' but in significant treatment effects for F_r2_' during the vegetative growth period (F(1,184) = 56.70, *p*<.001) and during senescence (F(1,171) = 14.00, *p*<.001). The development of F_r2_' included a more pronounced difference between treatments at the beginning of the season, a decline in mid-season, and an increase after heading, particularly under a[CO_2_].

## 4 Discussion

### 4.1 Abiotic environmental parameters

According to the German Meteorological Service (DWD), 2021 was an ambivalent year, generally following the long-term trends with slightly increased temperatures (Imbery et al., 2021). During the experiment, the vegetative growth period featured more favourable environmental conditions compared to the grain-filling phase, which faced a drought and heat period. The climatic conditions observed in 2021, characterised by increased temperatures, periods of drought, and episodes of heavy rainfall, align with future climate prediction models, increasing the relevance of our e[CO_2_] dataset for future projection scenarios.

The here described FACE setup has shown to be an effective experimental setup to increase the CO_2_ concentration in a winter wheat field. The system also managed to increase the [CO_2_] during cold and windy winter conditions. Outside the FACE operating hours, especially in the early morning hours, we recorded a substantial accumulation of the CO_2_ concentration in all sensors placed in the elevated and ambient CO_2_ ring. On windless summer nights, these values often exceeded thresholds of 1000 ppm. This effect could also be observed in various eddy-covariance stations (TERENO, https://ddp.tereno.net). The accumulation of CO_2_ can be explained by plant respiration mechanisms, which tend to increase with biomass accumulation over the season, peaking shortly before ear emergence and are generally higher during wind-still, warm nights with reduced air circulation (Ney and Graf, 2018; Pearman and Garratt, 2022). Additionally, soil respiration mechanisms of microorganisms releasing CO_2_ back into the atmosphere during the night contribute to the effect and may also lead to slightly increased values under e[CO_2_] (Lipson et al., 2005). Since the effect was observed in either treatment and mainly during the night when photosystems were idle, we presume that this effect had a neglectable influence on crop development in this study.

FACE experiments are constrained by their capacity for homogenous CO_2_ distribution, limiting the experimental area and leading to CO_2_ fluctuations. Although fluctuations in [CO_2_] are also present in natural environments, they are substantially greater in a FACE system. Allen et al. (2020) noted that these fluctuations can cause a reduced photosynthetic activity which can lead to an underestimation in yield, suggesting a yield data correction factor of 1.5. Furthermore, the authors argue that until the effects of fluctuating vs. constant elevated CO_2_ are better understood, modelling plant growth and yield will remain uncertain. The extent of these fluctuations can be largely managed through the design and technology of the FACE system. To ensure best possible results, the here presented FACE ring structure was limited to 18.5 m in diameter. The ability to achieve homogenous CO_2_ fumigation is further dependent on factors such as wind speed, direction and air temperature, which were carefully considered and accounted for in the design and execution of the experiment.

Despite the technical and financial challenges of CO_2_ fumigation, the target concentration was generally maintained at a high level. Although the scalability of FACE experiments is limited by these challenges, they offer the most accurate simulation of natural conditions and are therefore indispensable for understanding plant and ecosystem responses to future climatic conditions.

### 4.2 Yield parameters

Since 2021 has been a year with rather challenging weather conditions, and where extreme precipitation events led to increased lodging and harvest losses, our grain yields were compared to a multi-regional state cultivar trial. Despite the weather conditions, cultivars grown in the present study are in range with the state trial and obtained grain yields for plants grown at a[CO_2_] were marginally higher (+2.8%) (NRW Chamber of Agriculture, 2021).

The observed substantial increase in straw biomass under e[CO_2_] aligns with the prevailing favourable environmental conditions during the vegetative growth phase. This increase, coupled with the observed rise in photosynthetic efficiency, could contribute to more sustainable and multifunctional agricultural practice, where increased straw biomass not only contributes to carbon sequestration, e.g. carbon farming, but potentially also offers valuable ecosystem services or serves as a valuable industrial raw material. While the increase in photosynthetic efficiency correlates with biomass accumulation, it may not fully capture the complexity of the processes contributing to biomass accumulation. However, the synergy between the increased photosynthetic efficiency and the boosted growth in the early vegetative stage may be linked to clarify the potential shift in resource allocation as indicated by the Harvest Index (HI). Interestingly, Campesino was the only cultivar that maintained its grain-to-shoot biomass ratio. This cultivar belongs to the quality group B and is characterised by its low crude protein content. Moschus, an Elite-cultivar characterised by high crude protein content, had the highest decrease in the HI. Similar decreases in the HI have previously been reported in a meta-analysis on barley grown under elevated [CO_2_] (Gardi et al., 2022). The observed shift in the resource allocation for the large majority of the cultivars, favouring vegetative growth over investments into reproductive organs, is potentially based on the down-regulation of RuBisCo activity and electron transport under e[CO_2_] (Ainsworth et al., 2004). LIFT measurements performed during the grain filling phase showed an apparent down-regulation of the PSII operating efficiency in Moschus. In contrast, Campesino could maintain higher F_q_'/F_m_' during our last measurements under e[CO_2_]. Whether the higher CO_2_ concentration and the related change in the resource allocation of crops also led to an impairment of the crop quality needs further investigation.

Although a handful of FACE studies investigated the yield response of spring wheat, only very few studies explicitly focused on winter wheat. Dier et al. (2018) conducted a two-year FACE study with a single cultivar and observed an 18% increase in vegetative biomass and 17% in grain yield. In another experiment dealing with two winter wheat cultivars, conducted by Bunce (2012), no significant grain yield increase was detectable. The results obtained in our study indicated an above-average increase in vegetative dry matter for most of the cultivars. However, the mean grain yield was lower than in previous studies, except for the cultivars Asory and Campesino. The substantial relative increase in grain yield under e[CO_2_] observed for these two cultivars could potentially be attributed to genetic factors such as the integration of a rye translocation genome T1AL.1RS/T1RS.A1, which has previously been described to cope well with dry conditions during the grain filling stage (Dr. B. Hackauf, personal communication). The below-average grain yield increase could be attributed to the anthropogenic-caused rise in temperature, which is suggested to offset growth enhancements driven by e[CO_2_] as described in a recent meta-analysis (Helman and Bonfil, 2022). Disparities in reported biomass accumulation of other FACE studies can also be attributed to our selection of cultivars characterised by a very high yield, presumably reaching their maximum yield potential. The phenomenon known as yield plateauing may account for the non-environmentally caused limited increase under e[CO_2_]. Also, past studies often include fewer cultivars, typically with older release years. The diversity of these findings highlights the importance of studying cultivar-specific responses to e[CO_2_] and stresses the importance of genetic diversity in developing climate-resilient crops.

Both manual and UAV-based data demonstrated increased height for plants grown under e[CO_2_]. Intriguingly, this growth stimulation occurred early in the season and was maintained throughout the entire growth period, exhibiting only marginal variations. This trend indicates that the examined cultivars are especially prone to e[CO_2_] during early growth and that the investments made during that time were decisive for later development. Towards the end of the vegetation period, however, a decline of plant height was observed for plants grown under e[CO_2_], which may be linked to the observed phenomenon of earlier senescence, which was also noticeable in the EVI and in ChlF traits (see section 4.3 and 4.4). While a strong correlation was observed between manual plant height measurements and UAV-retrieved data, we also observed an increasing discrepancy between the two methods. The offset between the two methods can primarily be attributed to differences in the measurement technique. While manual measurements allowed for a simple determination of a plant’s tip, identifying the relatively small tips, i.e. ears, from a nadir perspective in UAV imagery with a resolution of several centimetres is challenging. The increasing disparities can also be attributed to observed alterations during the senescence phase. While ears maintained their height, leaves were shrinking and changing their angle, i.e., more downwards, leading to a potential decline in canopy height. Furthermore, during the processing of the UAV data, a 95% confidence interval was set to exclude extremes, e.g. overreaching plants. Similar observations were previously reported by Bareth et al. (2016). Although UAV-retrieved measurements seem to result in a slightly lower accuracy, they allow for large area assessments and thus are by far more time efficient.

### 4.3 Phenology

The potential to boost the vegetative growth by e[CO_2_] was also demonstrated by the significantly higher VI values (*p*<.001) observed under e[CO_2_] after canopy closure. As the vegetation period advanced, the EVI and OSAVI clearly responded to the disparities between a[CO_2_] and e[CO_2_] and were maintained up to anthesis. After that, we observed a noticeable decrease in VIs and photosynthetic performance measured by ChlF traits. This decline in photosynthetic activity is a result of the transition from the generative phase to the senescence phase, which is marked by a progressive browning of the vegetation. During the senescence period, the response was different for the different VIs. While EVI values for plants grown under e[CO_2_] slightly dropped below the values of plants grown under a[CO_2_], the NDVI values were maintained, suggesting that e[CO_2_] either extended the vegetation’s productive phase or mitigated the effects of senescence. On the other hand, the NDVI already showed rising values during the first flight campaign, suggesting that the increasing CO_2_ levels continued to have a consistent and prolonged influence on the plant height and biomass accumulation throughout the observation period and followed a similar trend as the ExG throughout the senescence period. The persistent increases in vegetation indices relative to ambient CO_2_ conditions indicate that the NDVI was generally less sensitive to increases in biomass, which has previously been documented (Baret and Guyot, 1991; Mutanga and Skidmore, 2004).

Often described as the final stage of a plant’s life cycle, senescence is an essential upcycling process where resources such as RuBisCo are reallocated within the plant to maximise reproductive success (Pyung et al., 2007). The visible degreening process or degradation of pigments results from oxidative stress, caused by an imbalance of the C/N ratio and is accompanied by reduced enzymatic activity, resulting in an increased H_2_O_2_ production (Agüera and De la Haba, 2018). To monitor senescence dynamics at the canopy level, temporally high-resolved data was recorded with PhenoCams. The ExG index, derived from the PhenoCam data, notably revealed a less pronounced senescence rate under e[CO_2_]. However, the declining ExG values may not solely be attributed to wilting leaves and chlorophyll degradation but also to an architectural change in the plants. This phenomenon is also evident in the UAV data (DOY 216), showing differences in canopy height between a[CO_2_] and e[CO_2_]. Even though one of the earliest visible signs of senescence is the breakdown of chlorophyll, leaf yellowing is not a good indicator of the early stages since it occurs when the process has proceeded for some time (Diaz et al., 2005). Bertheloot et al. (2008) described that senescence in winter wheat progresses from the bottom to the top of the canopy, influenced by the quantity of available protein in the vegetative organs. The instruments used in our study, PhenoCams and LIFT, captured this phenomenon differently due to their viewing angles. Specifically, PhenoCams, which capture the canopy from a side view, primarily focus on the upper leaves. In contrast, LIFT takes a nadir (top-down) view, giving it a more comprehensive look into the deeper layers of the canopy. This is crucial for understanding the differential effects of e[CO_2_] on the plant. Under e[CO_2_], the lower leaves, presumably richer in protein, show an earlier decrease in F_q_'/F_m_'. This suggests that resources are being reallocated to the upper leaves, allowing them to maintain higher photosynthetic activity for a longer period. Therefore, the combination of both systems under e[CO_2_] reflect not just current photosynthetic efficiency but also the plant’s adaptive resource allocation strategy in response to e[CO_2_].

### 4.4 Chlorophyll fluorescence traits

Regardless of the treatment, the obtained F_q_'/F_m_' parameter generally followed the expected trend of a high photosynthetic efficiency during the beginning of the vegetation period, followed by a decline over the vegetation period. This decline can be attributed to the natural ageing process of the plants, which consequently also leads to a continuous reduction of carbon fixation per unit leaf area throughout the growing season. Further examination of the data showed that all ChlF traits followed a consistent trend across varieties until the grain-filling stage. After DOY 170, however, the responses became strongly cultivar-specific.

The results of this study indicate that ChlF traits of plants grown under e[CO_2_] differed from those grown under ambient conditions. While under e[CO_2_], F_q_'/F_m_' was significantly increased during the vegetative period, it showed a more substantial decline towards the end of the vegetation period. Conversely, there was no discernible trend during the mid-season.

In this context, understanding the relationship between photosynthesis and sink capacity is critical. Existing research suggests that a plant’s ability to utilise excess assimilates produced under e[CO_2_] is likely driven by its sink capacity. Variations in the observed ChlF traits across different growth stages could reflect differences in sink strengths. Early in the growing season, resources are allocated primarily towards the developing stem and leaf tissues. The observed rapid growth under e[CO_2_] likely stimulated the production of NADPH and ATP, which are essential for efficient electron transport, thereby promoting photosynthetic efficiency. The gap between F_q_'/F_m_' under ambient and e[CO_2_] then narrows, possibly due to increased sink-driven respiration or potential acclimation effects (Lauriks et al., 2021).

However, as the plants approach heading and anthesis, the focus of resource allocation shifts from leaves to reproductive structures, such as developing inflorescence and grains, which serve as strong sinks. Although the timing of anthesis across different cultivars was relatively consistent and not significantly altered by e[CO_2_], the accelerated growth and increased carbohydrate production during the vegetative phase might indirectly lead to a more pronounced decline in F_q_'/F_m_' under e[CO_2_] conditions. The increased F_r1_' and F_r2_' values during the senescence phase suggest a slowing down of reoxidation rates under e[CO_2_], possibly due to a shift in resource allocation. Fewer electrons from PSII could be available for downstream processes like the Calvin cycle, leading to this deceleration in reoxidation rates. The strong correlation between F_r1_’ and F_r2_’ with F_q_’/F_m_’ indicates that e[CO_2_] likely influences the efficiency of the Calvin cycle, contributing to the observed changes in photosynthetic efficiency.

Correlation analysis of the ChlF traits and environmental factors revealed distinct correlations during different growth stages. During the vegetative growth phase, there is a positive correlation between temperature and F_q_'/F_m_' (*r* = .50), while the reoxidation efficiencies F_r1_’ and F_r2_’ exhibit a negative correlation (*r* = -.70 respectively *r* = -.44). The observed pattern in the vegetative phase could indicate that winter wheat optimises F_q_'/F_m_' under e[CO_2_], particularly at higher temperatures during the initial growth stages. A meta-analysis by Poorter et al. (2021) highlighted that the internal water-use efficiency of C_3_ plants tends to be significantly higher under e[CO_2_]. This could explain why winter wheat grown under e[CO_2_] maintained a relatively higher F_q_’/F_m_’, particularly during dry days.

When transitioning into the generative growth phase, a strong positive correlation exists between F_r2_’ and both temperature (*r* = .64) and PAR (*r* = .80), indicating increased electron transfer kinetics from the PQ pool to PSI. This may suggest an enhancement of reproductive growth processes. In the senescence growth phase, F_q_'/F_m_' is again positively correlated with temperature (*r* = .71) and PAR (*r* = .50), implying an augmented light-associated plant efficiency during the transition to senescence. In contrast, F_r1_' and F_r2_' exhibit a negative correlation with these factors during this phase (*r_T_*_emp_ = -.87 and *r_PAR_* = -.60, respectively *r_T_*_emp_ = -.60 and *r_PAR_* = -.40). These observations underline the intricate and dynamic interactions between photosynthetic efficiency, electron transfer kinetics, and environmental variables across the growth stages.

## 5 Conclusions

We demonstrated that a future increase in the atmospheric CO_2_ concentration, to an expected level of the second half of this century, significantly impacts the growth dynamics and development of modern winter wheat cultivars. Early vegetation stages particularly benefit from enhanced growth under e[CO_2_], a crucial phase where plants establish the foundation for subsequent development. However, e[CO_2_] also appears to alter the senescence process. This dual impact results in a changed resource allocation strategy, as evidenced by changes in yield parameters like the harvest index.

The observed variations in photosynthetic efficiency, quantified as F_q_'/F_m_', reflect a complex interplay of environmental conditions, developmental stages, and potentially genetic factors. This suggests that plants’ ability to exploit additional resources under e[CO_2_] may be constrained by varying sink capacities throughout the growth cycle. Such insights could guide targeted management interventions, such as the application of growth regulators or breeding programs aimed at optimising genetic composition for resilience under changing climatic conditions. Importantly, these findings were made possible by integrating an automated phenotyping platform in a FACE system in combination with an array of sensors. Platforms like this offer invaluable data for the assessment of climate-resilient crop cultivars. Moreover, continuous photosynthetic measurements could serve as a monitoring tool for assessing the impact of environmental stressors. This knowledge could then be applied to fine-tune crop management practices, enhancing yield while minimising the input, thereby contributing to broader efforts to make farming systems more sustainable.

## Data availability statement

The original contributions presented in the study are included in the article/**Supplementary Material**, further inquiries can be directed to the corresponding author.

## Author contributions

OK: Conceptualization, Data curation, Formal analysis, Investigation, Methodology, Project administration, Software, Visualization, Writing – original draft, Writing – review & editing. AC: Data curation, Formal analysis, Investigation, Writing – review & editing. JB: Data curation, Formal analysis, Investigation, Methodology, Software, Writing – review & editing. RP: Project administration, Resources, Writing – review & editing. EK: Conceptualization, Data curation, Formal analysis, Investigation, Methodology, Resources, Validation, Writing – review & editing. HP: Conceptualization, Methodology, Supervision, Validation, Writing – review & editing. UR: Conceptualization, Funding acquisition, Methodology, Project administration, Resources, Supervision, Writing – review & editing. OM: Conceptualization, Funding acquisition, Methodology, Project administration, Resources, Supervision, Validation, Writing – review & editing.
