# Supplementary material for: Field phenotyping of ten wheat cultivars under elevated CO2 shows seasonal differences in chlorophyll fluorescence, plant height and vegetation indices
Source: Front Plant Sci. 2024 Jan 8;14:1304751. doi: 10.3389/fpls.2023.1304751 (PMC10800489; doi:10.3389/fpls.2023.1304751)
Supplement: Supplementary file 1 [file DataSheet_1.docx]

## Data Availability Statement

The processed data is available on the Jülich DATA platform: <https://doi.org/10.26165/JUELICH-DATA/QJIY7C>

## Appendix

Table A1 Cultivar characteristics with German release years and phenological, yield and quality scoring under ambient CO_2_ conditions.

**Table A2:** Data acquisition time and environmental conditions during the field measurements.

**Table A3:** Absolute values for various yield parameters.

**
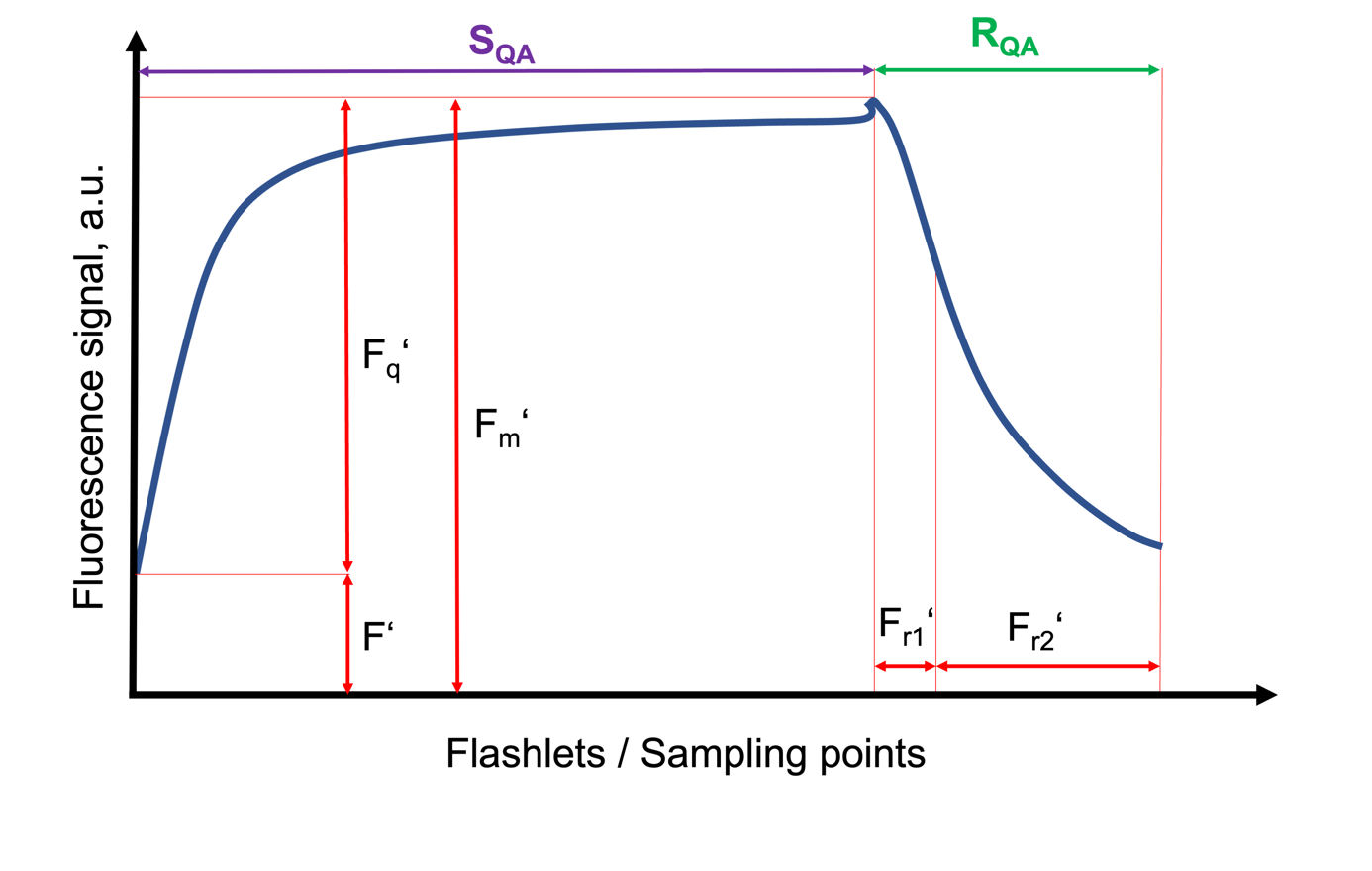
Figure A1:** Visualisation of an ideal LIFT transient with the saturation (S_QA_) and relaxation sequence (R_QA_) showing the initial (F'), maximal (F_m_') and variable fluorescence (F_q_') yield from light-adapted plants as well as the reoxidation efficiency of Q_A_¯ up to ~0.65 ms after F_m_' is reached, i.e., the kinetics of electron transfer from Q_A_ to PQ pool from light-adapted plants F_r1_' and the reoxidation efficiency of Q_A_¯ up to ~6.64 ms after F_r1_', i.e., the kinetics of electron transfer from PQ pool to PSI from light-adapted plants F_r2_'.


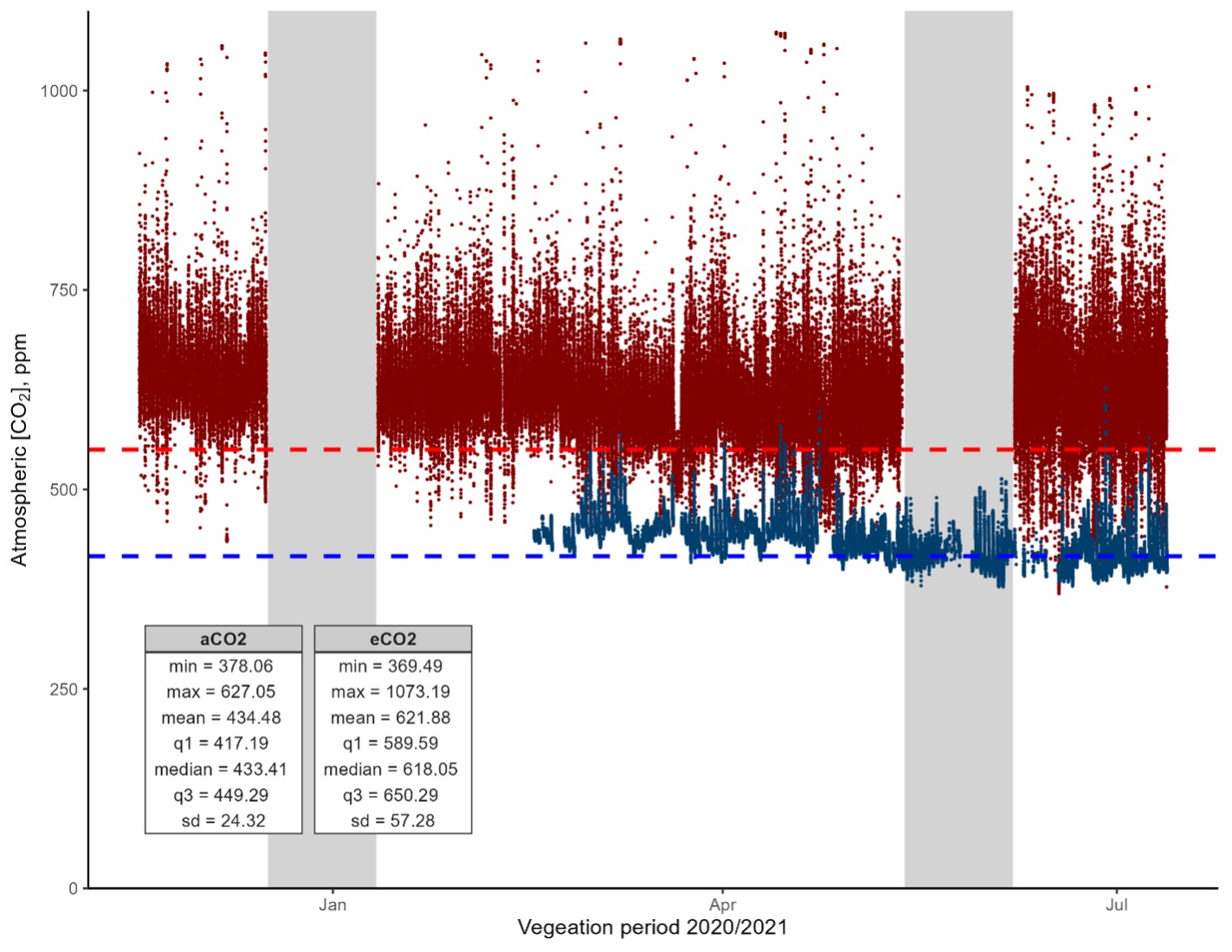


**Figure A2:** Mean ambient (~434 ppm) in blue and elevated (~622 ppm) atmospheric CO_2_ concentration in red, measured in the structures of the BreedFACE during the vegetation period in 2020/2021, from 8 a.m. to 5 p.m. Each dot represents the mean value of five measurements taken at ten-second intervals. The blue dashed line indicates the global mean annual CO_2_ concentration measured at the NOAA Global Monitoring Lab, Mauna Loa, [www.gml.noaa.gov](http://www.gml.noaa.gov). The red dashed line represents the target CO_2_ concentration (550 ppm). The left grey area indicates a safety break during the Christmas/New Year period when the system shut down for safety reasons, and the second indicates a period of unreliable elevated CO_2_ data due to an electrical problem.
